# Toward an integrated approach for mental health and psychosocial support and peacebuilding in North-East Nigeria: programme description and preliminary outcomes from ‘Counselling on Wheels’

**DOI:** 10.1192/bjo.2023.575

**Published:** 2023-10-12

**Authors:** Sharli Paphitis, Fatima Akilu, Natasha Chilambo, Abiye Iruayenama, Xena Samaroo, Asma'u Mustapha, Kimberley Goldsmith, Olawale Ismail, Petr Slovak, Eka Ikpe, Patrick Smith, Preeti Patel, Richard Sullivan, Melanie Abas, Funmi Olonisakin

**Affiliations:** Department of Health Services and Population Research, Institute of Psychiatry, Psychology and Neuroscience, King's College London, UK; and Department of Philosophy, Rhodes University, South Africa; Department of Psychology, Research and Policy, The NEEM Foundation, Abuja, Nigeria; and African Leadership Centre, School of Global Affairs, Faculty of Social Sciences and Public Policy, King's College London, UK; African Leadership Centre, School of Global Affairs, Faculty of Social Sciences and Public Policy, King's College London, UK; Department of Psychology, Research and Policy, The NEEM Foundation, Abuja, Nigeria; Department of Health Services and Population Research, Institute of Psychiatry, Psychology and Neuroscience, King's College London, UK; Department of Informatics, King's College London, UK; Department of Health Services and Population Research, Institute of Psychiatry, Psychology and Neuroscience, King's College London, UK; and Centre for Conflict and Health Research, King's College London, UK; Faculty of Life Sciences and Medicine, King's College London, UK

**Keywords:** Peacebuilding, mental health and psychosocial support, violent extremism, low- and middle-income countries, psychosocial interventions

## Abstract

**Background:**

Despite theoretical support for including mental health and psychosocial support (MHPSS) with peacebuilding, few programmes in conflict-affected regions fully integrate these approaches.

**Aims:**

To describe and assess preliminary outcomes of the Counselling on Wheels programme delivered by the NEEM Foundation in the Borno State of North-East Nigeria.

**Method:**

We first describe the components of the Counselling on Wheels programme, including education and advocacy for peace and social cohesion through community peacebuilding partnerships and activities, and an MHPSS intervention open to all adults, delivered in groups of eight to ten people. We then conducted secondary analysis of data from 1550 adults who took part in the MHPSS intervention, who provided data at baseline and 1–2 weeks after the final group session. Vulnerability to violent extremism was assessed with a locally developed 80-item scale. Symptoms of common mental disorders were assessed with the Depression, Anxiety and Stress Scale (DASS-21) and Post-Traumatic Stress Disorder Scale (PTSD-8). Data were analysed through a mixed-effect linear regression model, accounting for clustering by community and adjusted for age and gender.

**Results:**

After taking part in group MHPSS, scores fell for depression (−5.8, 95% CI −6.7 to −5.0), stress (−5.5, 95% CI −6.3 to −4.6), post-traumatic stress disorder (−2.9, 95% CI −3.4 to −2.4) and vulnerability to violent extremism (−44.6, 95% CI −50.6 to −38.6).

**Conclusions:**

The Counselling on Wheels programme shows promise as a model for integrating MHPSS with community peacebuilding activities in this conflict-affected region of Africa.

Peacebuilding contains, creates and sustains the breadth of processes, approaches and phases needed to move conflict toward more sustainable and peaceful relationships.^1^ Peacebuilding activities address structural problems, the social dynamics of building relationships and the development of a supportive scaffolding for peace. It may use techniques such as reconciliation through mutual acceptance between groups post-conflict; transitional justice through criminal prosecutions, memorialisation and symbolic reparations; and repairing and building the social fabric.^1,2^ There is increasing theoretical agreement that peacebuilding activities should also include mental health and psychosocial support (MHPSS) to ensure effective social change.^2^ MHPSS is widely recognised as important for conflict situations because of the short- and long-term effects of the exposure of war and traumatic events.^2^ Meta-analysis of epidemiological data suggests that 17.3% of people living in conflict settings meet the criteria for depression, and 15.4–22% meet the criteria for post-traumatic stress disorder (PTSD).^3,4^ Systematic review evidence suggests that there are few examples of fully integrated MHPSS and peacebuilding programmes, and a weak evidence base for the effects and outcomes of an integrated approach to MHPSS and peacebuilding.^5^ Historically, the impact of armed conflict on mental health has been addressed on an individual psychological level, through MHPSS.^2^ However, thorough attempts to integrate MHPSS and peacebuilding efforts have increased attention on wider social experience (relationships, values and culture) of peacebuilding for both individual transformations and as the foundation for community-level resilience, conflict resolution capacity-building and social cohesion.^6–8^ There is a gap in our understanding of the nature and impact of integrated programmes for internally displaced persons, i.e. people who are forced to move from their home because of humanitarian crisis, without crossing international borders. Here, we outline the Counselling on Wheels programme delivered by the NEEM Foundation in the Borno State of North-East Nigeria, in which MHPSS and peacebuilding techniques are systematically integrated to improve mental health outcomes and reduce vulnerability to violent extremism. We describe the approach and the activities of the peacebuilding arm. We present data from a pre–post study of the impact of the Counselling on Wheels programme on reducing vulnerability to violent extremism and improving mental health outcomes, specifically depression, anxiety, stress and PTSD.

## Method

### Partnerships between The NEEM Foundation and the Borno communities

The Borno region in North-East Nigeria comprises over 40 ethnic groups, with the largest being Kanuri. In 2016, the Borno State's projected population was 5.9 million people; however, the true figure is likely to be higher because of the large number of internally displaced persons living in the region.^9^ The Boko Haram conflict has caused a humanitarian crisis and displacement in North-East Nigeria over the past decade, and has been classed as ‘one of the most pronounced, multifaceted and complex humanitarian crises known to the international community’.^10^ The NEEM Foundation is a crisis response non-governmental organisation founded in 2016. Their mission is to strengthen the resilience and capacity of crisis-affected communities across Nigeria and the Lake Chad Basin. They design and deliver reintegration and stabilisation services that target displaced communities, as well as former associates of violent armed groups. Key approaches include education, psychosocial support and advocacy. The Counselling on Wheels programme sits in the MHPSS arm of the NEEM Foundation. In 2020, Counselling on Wheels worked with six communities across the Borno region in Bintu Suga, Injin Kusa, Ngomari Ndalori, Muna Ethiopia, Bolori Burin and Bolori Shuwari.

#### The NEEM Foundation's approach

Survivors of the violent insurgency in North-East Nigeria often have multiple layers of trauma. The negative effect of this subjective internal traumatic reality often has undesired consequences on an outward and social approach toward peacebuilding: trauma amplifies the risk of adverse social patterns such as drug and substance misuse, gender-based violence and organised criminality. The emergence of these fracture a sense of morality, and ethical considerations become flexible and transactional in such settings. This impedes the peacebuilding process. Therefore, the NEEM Foundation's approach to extrinsic peacebuilding and social cohesion starts by first addressing hidden and intrinsic emotional and cognitive challenges brought on by the conflict.^11^

To deliver the Counselling on Wheels programme in these communities, the NEEM Foundation builds relationships with community *Bulamas* (traditional leaders) through community liaison officers. Community liaison officers introduce community leaders to the Counselling on Wheels programme by using an asset-based approach, providing community stakeholders with an opportunity to highlight their own solutions to improving peacebuilding and mental health challenges in the community, as well as how these can be incorporated into the programme. Working with the community liaison officers, community leaders gain an in-depth understanding of the MHPSS and peacebuilding activities that will be offered by the NEEM Foundation within their community. Community liaison officers incorporate psychoeducation within their meetings with community leaders to address the stigma of visiting mental health professionals and to develop the understanding that mental health is as important as physical health. The partnerships developed between the community liaison officers and community leaders mean that the NEEM Foundation's programmes are contextually responsive to specific community's assets and needs, and, importantly, that community leaders work with the NEEM Foundation to legitimise and advocate for the MHPSS and peacebuilding activities offered within their communities.

### Community-level peacebuilding

**Fig. 1:**
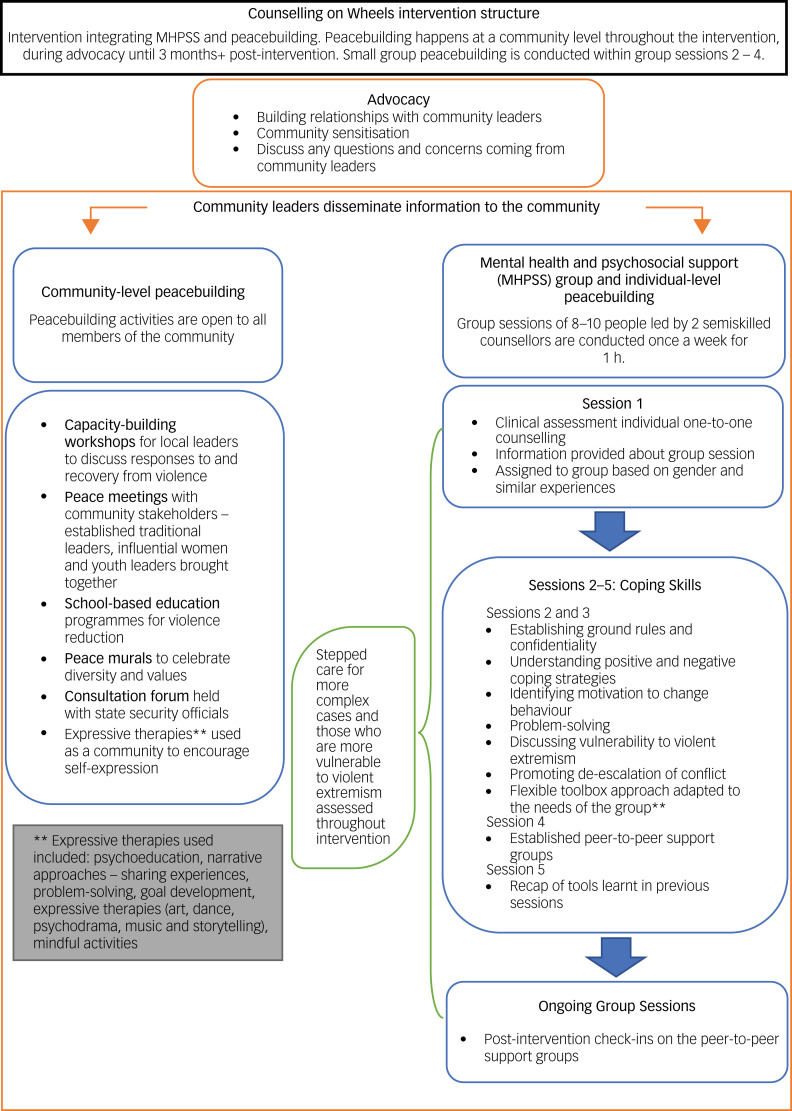
Counselling on Wheels intervention structure.

Peacebuilding activities facilitated by psychosocial workers were open to all members of each community. The workers, recruited based on their skills and knowledge of psychosocial support, understanding of the local culture and ability to communicate in the local language, participated in a 2-week intensive training course on peacebuilding activities. At the community level, peacebuilding activities incorporated five different modalities (see Fig. 1). Peace mechanisms in communities were encouraged through ‘peace meetings’ with various community stakeholders. A bottom-up holistic approach to peacebuilding in the community was used to resolve community grievances and encourage social cohesion. Capacity-building workshops for local leaders were conducted to train the community leaders in peacebuilding techniques to address vulnerability to violent extremism through adopting the use of tolerance and forgiveness, and reducing prejudice, stereotypes and stigma. Consultation forums discussing soft-power approaches to tackling insecurity were held with local security officials and agencies. The NEEM Foundation also established partnerships with schools to deliver educational activities, including programmes that specifically focused on discouraging and preventing gender-based violence. Finally, peace murals – walls in which community members were encouraged to express messages of peace and tolerance – were created to celebrate diversity and affirm shared community values.

### MHPSS groups and individual-level peacebuilding

The first stage of counselling support involved a one-to-one clinical session with community members, during which demographic variables, personal history, psychological symptoms and vulnerability to violent extremism assessments were taken. Following clinical assessment, participants were assigned groups according to gender, psychological symptoms and experiences. Those presenting with a higher psychological risk and those more vulnerable to violent extremism were provided with subsequent one-to-one sessions between their group work, and this evaluation continued throughout the intervention process.

The therapeutic intervention followed a three-session protocol following clinical assessments (see Fig. 1 for content of group intervention). Each session lasted an hour, and was conducted once a week in groups of eight to ten people. This was a task-shifted intervention led by two semi-skilled counsellors. Counsellors participated in a 2-week intensive training course in delivering mental health therapy, understanding trauma, safety and assessment. Counsellors received ongoing training and mentoring 1 day per week from The NEEM Foundation's senior psychologists.

A flexible toolbox approach, according to the presenting symptoms, gender, experiences and circumstances, was used to inform the session protocol. The three sessions combined psychoeducation (on symptoms of emotional distress as well as the effects of emotions, thoughts and beliefs on behaviour), with narrative approaches (allowing recipients to share their stories and experiences in a safe and confidential space). Expressive therapies were incorporated, giving recipients a space for self-expression where talking about experiences may have been too difficult. Group work included developing positive coping strategies and learning relaxation techniques. Groups explored their motivations to change their behaviours and reviewed techniques used to develop behavioural change.

Throughout the MHPSS group intervention, there was a strong focus on combined psychosocial and peacebuilding elements, including strategies to de-escalate conflict, and build resilience at a personal, family and community level. Participants were encouraged to recognise what makes them and their community resilient, as well as how they marshal both internal and interpersonal resources to cope with and manage problems.

At the final stage of the counselling sessions, one-to-one assessments were conducted. Stepped care is provided to those with ongoing severe presenting symptoms; those recipients are referred to a psychologist. After the completion of the final session, community mental health support groups were composed. The group structure of the therapy easily translates into a supportive group model that allows recipients to continue groups using a peer-to-peer support structure, with lead peer facilitators selected through a voting process within the groups themselves. Group leaders receive ongoing mentoring and support from the NEEM Foundation's counsellors. At 2–3 months post-intervention, the NEEM Foundation team visited communities to provide ongoing support to community leaders and peer groups.

### The Counselling on Wheels programme

Between August 2020 and January 2021, 2283 participants took part in the Counselling on Wheels programme, from six communities in the Borno State of North-East Nigeria. Information about, and invitations to participate in, peacebuilding and MHPSS activities was disseminated as widely as possible across whole communities by community liaison officers, community leaders and independent community representatives. All community members were made aware that participation was voluntary and that no material support would be provided. All participants included in this study were over 18 years of age. The intervention took place over a period of approximately 7 weeks.

Secondary analysis from a single-arm, pre–post observational study examining the effect of the Counselling on Wheels programme on mental health outcomes and vulnerability to extremism was conducted. This was measured on two occasions: at baseline (1 week pre-intervention; time point 1) and at 1–2 weeks after the final session (time point 2).

Ethical approval for the secondary analysis of these data in the current evaluation was granted by the London School of Hygiene and Tropical Medicine (approval number 25581). The Counselling on Wheels programme was conducted with permission from the Borno State Humanitarian Response Committee. The purpose of the intervention was explained during assessment, and recipients consented to engagement and data collection verbally, because of literacy issues. Recipients’ data was deidentified for analysis, to preserve confidentiality.

#### Measures

Clinical outcomes on depression, anxiety and stress were measured with the Depression, Anxiety and Stress Scale (DASS-21).^12^ The DASS-21 is a 21-item self-report questionnaire that has been shortened from the DASS.^13^ DASS-21 has been a widely accepted, reliable and valid measure of symptoms of depression, anxiety and stress.^14–17^ It has also been endorsed for use in Nigeria, with Cronbach's alpha values of 0.81, 0.89 and 0.78 for depression, anxiety and stress subscales, respectively.^18,19^ Scores are summed for each subscale, and the total for each scale is multiplied by 2. Post-traumatic stress symptoms were measured by the brief version of the Post-Traumatic Stress Disorder Scale (PTSD-8).^20^ The PTSD-8 is an eight-item self-report assessment tool derived from the Harvard Trauma Questionnaire (HTQ), assessing PTSD symptoms such as intrusion, avoidance and hypervigilance. The HTQ has previously been used in northern Nigerian populations,^21^ and has demonstrated some cross-cultural validity,^22^ including other sub-Saharan African settings.^23,24^ The PTSD-8 is a reliable tool, with a Cronbach alpha level of 0.84,^25^ and has been widely used with displaced populations.^26–28^ The PTSD-8 was not developed with a fixed cut-off point, but previous Counselling on Wheels analysis used a score of ≥17 to indicate probable PTSD.

The Vulnerability to Violent Extremism Scale (VVES)^11^ is a secondary measure used to assess vulnerability to violent extremism, developed by the NEEM Foundation. NEEM Foundation researchers, including a forensic clinical psychologist (F.A.) trained in both Nigeria and the UK, developed the VVES to rate internal and external vulnerabilities to violent extremism, and protective factors. VVES is based on risk assessment tools such as the violent extremism risk assessment protocol.^29^ The NEEM Foundation developed the VVES to inform the Counselling on Wheels programme by identifying areas where individuals could build resilience.

The VVES is an 80-item self-report assessment organised into three categories: internal vulnerabilities (attitude, ideology, belief, grievance, vengeance, intent, affect, suicidal ideation), external vulnerabilities (social factors, economic factors, context, history, identity, capacity, cognitive style) and protective factors (e.g. strong social support or strong attachment and bonds).^11^ The VVES is scored on a five-point Likert scale from 1 (‘strongly disagree’) to 5 (‘strongly agree’). Scores were categorised as low vulnerability (80–159), moderate vulnerability (160–319) and high vulnerability (320–400). Examples of items include ‘we need to fight violence with violence’ (attitude), ‘I feel humiliated’ (affect) and ‘people around me support the fighting’ (context).^11^

### Statistical analysis

Descriptive statistics were provided for sociodemographic variables and intervention outcomes. To evaluate the effect of the intervention, mixed-effect linear regressions were conducted on the pre- and post-intervention mental health outcome scores and vulnerability to violent extremism scores, where both pre and post measures were dependent variables, age and gender were included as independent variables, and a random intercept to account for the variation between participants within the six community clusters mentioned.

## Results

Overall, 2283 recipients underwent clinical assessment to participate in group MHPSS and individual-level peacebuilding activities. Errors in collection led to a significant amount of data being excluded; 851 points were excluded because of missing identification numbers and 221 data pairs were excluded because of missing demographic variables. The final cleaned data-set provided complete and accurate data on 1550 recipients.

### Demographic variables

Analysis of the 1550 recipients indicated that, at baseline, 88% of recipients were women and 12% were men. Most recipients (58%) were aged between 18 and 35 years, 24% were aged between 36 and 45 years and 19% were aged ≥46 years. There was a high level of ethnic diversity in the sample, with participants identifying as Kanuri (50%), Hausa (14%), Shuwa (11%) and Fulani (6%). Ethnicities with fewer than 1% of recipients were combined into an ‘other ethnicities’ category for analysis. Most recipients were married (76%), unemployed (97%) and had no formal education (78%) (see Supplementary Table 2 available at https://doi.org/10.1192/bjo.2023.575 for demographic variables).

### Mental health and vulnerability to violent extremism

Table 1 presents the descriptive analysis that was conducted on pre- and post-intervention outcome measures. Unadjusted mean scores in all measures were lower post-intervention than pre-intervention. An error in data entry resulted in three depression scores being excluded from the analysis.

**Table 1:** Outcome measures pre-and post-adjusted mean difference (*N* = 1550)

Outcome measure (possible score)	Pre-intervention time point 1, mean (s.d.)	Post-intervention time point 2, mean (s.d.)	β1	*P*-value
DASS-21 (depression) (0 to ≥28)	21.8 (12.1)	16.0 (12.7)	−5.8 [95% CI −6.7 to −5.0]	<0.001
DASS-21 (anxiety) (0 to ≥20)	21.7 (11.9)	15.9 (12.5)	−5.8 [95% CI −6.7 to −5.0]	<0.001
DASS-21 (stress) (0 to ≥34)	22.7 (11.9)	17.3 (12.8)	−5.5 [95% CI −6.3 to −4.6]	<0.001
PTSD-8 (8–32)	17.4 (6.7)	14.5 (7.5)	−2.9 [95% CI −3.4 to −2.4]	<0.001
VVES (80–400)	209.0 (86.0)	165.3 (86.7)	−44.6 [95% CI −50.6 to −38.5]	<0.001

DASS-21, Depression, Anxiety and Stress Scale; PTSD-8, Post-Traumatic Stress Disorder Scale; VVES, Vulnerability to Violent Extremism Scale.

There was evidence of a significant decrease of more than 5 points in scores for depression (β1 = −5.8, 95% CI −6.7 to −5.0; *P* < 0.001), anxiety (β1 = −5.8, 95% CI −6.7 to −5.0; *P* < 0.001) and stress (β1 = −5.5, 95% CI −6.3 to −4.6; *P* < 0.001), after the intervention. There was also a significant decrease of more than 2 points in PTSD scores between pre- and post-intervention (β1 = −2.9, 95% CI −3.4 to −2.4; *P* < 0.001). Finally, there was also a significant decrease of more than 40 points in scores from the VVES assessment after the intervention was applied (β1 = −44.6, 95% CI −50.6 to −38.6; *P* < 0.001).

The model was adjusted for age, gender, ethnicity, majority ethnic status and majority language status (see Supplementary Table 3). These variables had little effect on the intervention outcomes, and the changes in scores remained significant after these variables were controlled for.

## Discussion

Our results support previous findings in the grey literature that the Counselling on Wheels programme in North-East Nigeria is promising as an integrated peacebuilding and MHPSS group intervention.^11^ Although there is an evidence base for MHPSS initiatives in humanitarian crisis settings, few integrate MHPSS with peacebuilding.^2^ Joint World Health Organization and United Nations programmes providing mental health services to people affected by the conflict have recently launched in Somalia and Burkina Faso.^30^ Similar programmes to Counselling on Wheels, delivering ten sessions of cognitive–behavioural therapy,^31^ individual and group counselling services,^32^ and community-based sociotherapy,^33^ have been implemented in Uganda, Burundi and the Democratic Republic of Congo with mixed results. Scholars and practitioners at the MHPSS and peacebuilding nexus continue to emphasise the need for more robust research at this intersection.^2^ Results from the secondary analysis of the 2020 Counselling on Wheels programme show a significant positive effect on all outcome variables, which maintained its significance even after controlling for sociodemographic factors such as age, gender, ethnicity, majority ethnic status and majority language status. Data for this analysis were collected in a real-world situation by an non-governmental organisation in Nigeria, and thus has high levels of ecological validity that helps to bolster confidence that the intervention can be effective in the complex and challenging situations it was designed for. Furthermore, using a toolbox approach, this intervention was able to cater to the needs of the individuals in the group as opposed to using a ‘one size fits all’ approach. This may have increased the effectiveness of the intervention, and future studies should investigate the most beneficial mechanisms within the intervention. This is particularly encouraging for developing integrated peacebuilding and MHPSS initiatives, especially for those that aim to both improve the mental health of communities and reduce vulnerability to violent extremism in conflict-affected zones, where individuals are experiencing high levels of trauma and psychosocial need, with little access to psychological support.^34,35^

Important differences in demographic variables in our analysis are worth noting. Women were overrepresented in the intervention (88%) in the Counselling on Wheels programme, compared with 48% in the overall Borno population.^36^ This trend is not uncommon outside of North-East Nigeria, with cultural barriers and masculine stereotypes affecting male engagement in psychosocial programmes.^37^ However, men who did engage in the intervention were equally likely to have completed the treatment as women in the study.

### Limitations

The pre–post design with no control group means that we do not know if mental health improvement might have occurred without the group therapy, as a result of regression to the mean/natural improvement over time. However, this sort of design is common for evaluations conducted in humanitarian settings, where the use of a control group is often seen as unacceptable or unethical.^38^ The duration of follow-up was limited to shortly after four sessions of therapy; thus, we cannot comment on longer-term outcomes. Measures were administered through the counsellors rather than self-reports because of high rates of illiteracy in the population. This can increase the chances of demand characteristics, which may have affected internal validity. The DASS-21 and PTSD-8 were not culturally adapted, and their translation from English to other languages for non-English speakers was conducted verbally *ad hoc* by counsellors. Additionally, the VVES is not a validated questionnaire. Counterextremism is a novel field and measurement remains a debated topic, with no commonly agreed metric.^39^

A total of 24% of the data was excluded because of data entry issues. This is a common in mobile interventions, as well as when data is collected in contexts with poor electricity and connectivity, and high levels of relocation.^40^

Importantly, although this programme has shown preliminary validity in this setting, the complexities of other conflict ecosystems means that it is important that adapted programmes of this type are properly tested and adapted for local contexts, with community involvement.

In conclusion, the Counselling on Wheels programme demonstrates that peacebuilding and MHPSS can be integrated in a systematic way, emphasising the importance of community partnerships at multiple levels for the effective delivery of a holistic approach to peacebuilding and MHPSS. The analysis presented contributes to the evidence base for the impact and outcomes of an integrated approach to MHPSS and peacebuilding. We conclude that Counselling on Wheels is a promising grassroots, bottom-up programme integrating MHPSS and peacebuilding that can serve as a model for future programmes attempting to integrate MHPSS and peacebuilding to improve mental health and reduce the risk of relapsing into conflict through building resilience. Future research should be conducted on the mechanisms behind peacebuilding activities integrated into MHPSS programmes to further understand pathways to decrease vulnerability to violent extremism.

## Supporting information

Paphitis et al. supplementary material Paphitis et al. supplementary material

## Data Availability

The data that support the findings of this study are available from the corresponding author, N.C., upon request.
