# Supplementary material for: Toward an integrated approach for mental health and psychosocial support and peacebuilding in North-East Nigeria: programme description and preliminary outcomes from ‘Counselling on Wheels’
Source: BJPsych Open. 2023 Oct 12;9(6):e190. doi: 10.1192/bjo.2023.575 (PMC10594183; doi:10.1192/bjo.2023.575)
Supplement: Paphitis et al. supplementary material [file S2056472423005756sup001.pdf]

Table 2: Socio-demographic characteristics of recipients included in final analysis(N=1550)

Demographic Characteristic	Frequency(%[a])
**Sex**	
**Female**	1370(88)
**Male**	180(12)
**Age**	
**18-20**	213(14)
**21-25**	183(12)
**26-30**	296(19)
**31-35**	204(13)
**36-40**	254(16)
**41-45**	112(7)
**46-50**	145(9)
**51-55**	45(3)
**56-60**	56(4)
**61+**	42(3)
**Ethnicity**	
**Kanuri**	777(50)
**Hausa**	218(14)
**Shuwa**	172(11)
**Fulani**	95(6)
**Babur**	42(3)
**Marghi**	40(3)
**Mafa**	40(3)
**Gwoza**	27(2)
**Bura**	24(2)
**Karekare**	23(2)
**Other ethnicities**	92(6)
**Relationship Status**	
**Divorced**	19(1)
**Married**	1173(76)
**Separated**	19(1)
**Single**	162(11)
**Widowed**	177(11)
**Employment Status**	
**Yes**	49(3)
**No**	1501(97)
**Education Status**	
**No Formal education**	856(55)
**Non-Western Education**	506(33)
**Western Education**	188(12)

[a]may not sum to 100% due to rounding
